# Development of Myelodysplastic Syndrome in a Patient With Pernicious Anemia During the Course of Replacement Treatment

**DOI:** 10.7759/cureus.64650

**Published:** 2024-07-16

**Authors:** Yoshiki Uemura, Kazuto Togitani, Mayuko Kitaoka, Keitaro Yano, Mitsuo Okada

**Affiliations:** 1 Department of Hematology, Chikamori Hospital, Kochi, JPN; 2 Department of Gastroenterology, Chikamori Hospital, Kochi, JPN

**Keywords:** pernicious anemia, chromosome abnormalities, wilm’s tumor-1 mrna, myelodysplastic syndrome (mds), megaloblastic anemia

## Abstract

Megaloblastic anemia (MBA) is a reversible metabolic disorder that responds well to vitamin B12 supplementation. It contrasts with myelodysplastic syndrome (MDS), an irreversible neoplastic condition characterized by hematopoietic stem cell abnormalities. To date, no association has been identified between these two distinct etiologies, and they are considered independent diseases. However, despite their distinct classifications, both conditions present macrocytic anemia, similar bone marrow findings, and sometimes have common chromosomal abnormalities, which can lead to occasional misdiagnoses. Herein, we present a patient initially diagnosed with pernicious anemia (PA) who showed improvement with replacement therapy but subsequently became resistant to treatment and eventually developed MDS. Quantitative assessment of Wilm’s tumor-1 (WT1) mRNA has emerged as a valuable tool for gauging MDS disease status and distinguishing it from related disorders, such as aplastic anemia. In our investigation of 30 patients with MBA, we explored WT1 mRNA expression. We observed its presence in 10 patients with PA, which suggests a potential link between PA and hematopoietic tumors.

## Introduction

Megaloblastic anemia (MBA) manifests as dysplastic and ineffective hematopoiesis in bone marrow morphology [1,2] and occasionally exhibits a chromosomal karyotype that is also found in myelodysplastic syndrome (MDS) and acute myeloid leukemia (AML) [3]. Thus, these conditions can often be confused. Nonetheless, the clinical findings, cell morphology, and abnormal chromosomal karyotype of MBA show substantial normalization in response to replacement therapy. As a result, MBA and MDS are deemed mutually exclusive. To date, only a single case of concurrent onset of pernicious anemia (PA) and MDS has been reported in the literature [4]. In that case, although the patient’s macrocytosis and neurological symptoms abated with replacement therapy, the anemia, triple-lineage bone marrow dysplasia, and persistent chromosomal abnormalities eventually progressed to AML.

Patients with MBA occasionally exhibit chromosomal abnormalities similar to those indicative of MDS [5]. Quantification of Wilm’s tumor-1 (WT1) mRNA is a valuable indicator of disease status for both MDS and AML [6]. In one study, WT1 mRNA was quantified at the time of diagnosis in 30 patients with MBA, and its expression was detected in 10 of these patients. That study was the first to investigate WT1 mRNA expression in MBA.

Herein, we present a rare case of PA that developed resistance to replacement therapy, suggesting a potential transition to MDS. Through literature review and analysis of the correlation between clinical course and WT1 mRNA expression, we report the possibility of overlap in the pathologies of MBA and MDS.

## Materials and methods

Study design

In this case-control study, we included 30 patients with MBA (14 males and 16 females, aged 57-91 years) at our hospital between 2017 and 2023. This study was approved by the institutional ethics committee of Chikamori Hospital (approval no.: 323).

Chromosomal analysis of bone marrow cells was performed in 27 of the patients. The patients were offered anti-intrinsic factor antibody (IFA) testing via the immunoassay method at their own expense, to which 27 patients consented. At the initial consultation, WT1 mRNA was quantified via real-time PCR in all the patients, to differentiate them from those with MDS.

Inclusion criteria

We retrospectively identified all patients diagnosed with MBA based on the following criteria. All of them had macrocytic anemia with hemoglobin (Hb) levels < 10 g/dL, mean corpuscular volume (MCV) > 120 fL, vitamin B12 levels < 100 pg/mL, and increased megaloblasts, as revealed through bone marrow aspiration. Replacement therapy with intramuscular injection of vitamin B12 was performed, and all the patients’ Hb levels recovered to ≥ 10 g/dL.

## Results

Among the entire patient cohort, 23 tested positive for anti-IFA, four tested negative, and three were not tested. Quantification of WT1 mRNA before replacement therapy revealed elevated levels in 10 of the patients - eight of whom were anti-IFA-positive, whereas the remaining two were not tested. Following replacement therapy, WT1 mRNA levels were normalized in seven of the 10 patients who tested positive; the remaining three were not followed up. Chromosomal abnormalities were observed in five of the patients before replacement therapy; among them, two were anti-IFA-positive, two were anti-IFA-negative, and one was not tested. An increase in WT1 mRNA levels was noted in two of the five patients with chromosomal abnormalities. After replacement therapy, the chromosomal abnormalities disappeared in two of the patients, although the statuses of the other three remained unknown, as they were not followed up (Table 1). Replacement therapy successfully improved anemia in all 30 of the patients.

**Table 1:** Thirty cases of megaloblastic anemia encountered in our department (2017–2023) IFA, intrinsic factor antibody; WT1, Wilm’s tumor-1; pre, pre-treatment; post, post-treatment; NEG, negative; PO, positive; N/A, not applicable

Case	Age	Sex	IFA	WT1 mRNA (pre)	WT1 mRNA (post)	Chromosome (pre)	Chromosome (post)
1	72	♂	NEG	< 50	N/A	t(11;17)(q21)	46, XY
2	66	♂	NEG	< 50	N/A	46, XY	N/A
3	74	♂	N/A	< 50	N/A	46, XY	N/A
4	85	♂	PO	1.7 × 10^2^	< 50	46, XY	N/A
5	64	♀	PO	< 50	N/A	46, XX	N/A
6	89	♀	PO	< 50	N/A	46, XX	N/A
7	92	♂	PO	< 50	N/A	46, XY	N/A
8	82	♀	PO	1.8 × 10^2^	< 50	47, XX+Y, 47, XX, +8	46, XX
9	75	♂	NEG	< 50	N/A	46, XY	N/A
10	96	♂	PO	< 50	N/A	45, X-Y	N/A
11	73	♂	PO	71	< 50	46, XY	N/A
12	72	♀	PO	< 50	N/A	46, XY	N/A
13	77	♂	NEG	< 50	N/A	57, XX, -6, +16, +17, 18+18, +19, +20, +6mar	N/A
14	73	♂	N/A	84	< 50	N/A	N/A
15	74	♀	PO	< 50	N/A	N/A	N/A
16	82	♀	PO	< 50	N/A	46, XX	N/A
17	81	♂	PO	< 50	N/A	46, XY	NA
18	87	♀	PO	< 50	N/A	46, XX	N/A
19	84	♀	PO	< 50	N/A	46, XX	N/A
20	59	♂	PO	< 50	N/A	46, XY	N/A
21	57	♀	PO	< 50	N/A	46, XX	N/A
22	74	♂	N/A	77	N/A	46, XY, del(9)(q?)	N/A
23	80	♂	PO	1.2 × 10^2^	< 50	46, XY	N/A
24	78	♀	PO	2.0 × 10^2^	< 50	46, XX	N/A
25	60	♀	PO	1.9 × 10^2^	N/A	46, XX	N/A
26	80	♀	PO	< 50	N/A	46, XX	N/A
27	91	♀	PO	< 50	N/A	N/A	N/A
28	76	♀	PO	1.7 × 10^2^	N/A	46, XX	N/A
29	85	♀	PO	< 50	N/A	46, XX	N/A
30	87	♀	PO	2.3 × 10^2^	< 50	46, XX	46, XX, ?inv(2)(p21q21)
							47, XX, t(6:11)(q21:q23.3), +15

**Figure 1:**
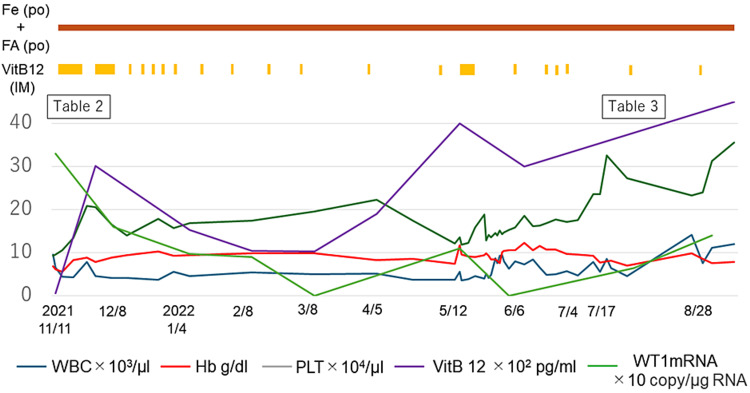
Clinical course of the patient Fe (PO), sodium ferrous citrate tablets 50 mg/d (per os); FA (PO), folic acid 100 mg/d (per os); VitB12 (IM), vitamin B12 (intramuscular injection) 500 µg/d; WBC, white blood cell; Hb, hemoglobin; PLT, platelets

However, one patient - an 87-year-old woman (case 30) - experienced a relapse of her anemia despite receiving continuous replacement therapy (Figure 1).

She presented with macrocytic anemia, chronic thrombocytopenia, and anorexia, and she had no prior history of malignant disease, anticancer drug administration, or radiation exposure. Her complete blood count revealed a white blood cell count of 9,600/µL, Hb level of 6.8 g/dL, MCV of 132.2 fL, and a platelet count (PLT) of 9.2 × 10^4^/µL. A peripheral blood smear indicated the presence of hyperlobulated neutrophils. Biochemical tests showed her vitamin B12 level at 57 pg/mL and folic acid level at 2.1 ng/mL. Bone marrow aspiration revealed giant metamyelocytes/stab cells, erythroid hyperplasia, megaloblastic changes, and isolated multinucleated megakaryocytes. Her chromosomal karyotype was normal, but her WT1 mRNA level was elevated to 2.3 × 10^2^ copies/µg RNA (Table 2). Due to her positive anti-IFA antibody status, she was diagnosed with PA. 

**Table 2:** Patient laboratory data upon admission WBC, white blood cell count; RBC, red blood cell count; Hb, hemoglobin; Ht, hematocrit; MCV, mean corpuscular volume; PLT, platelet; TP, total protein; ALB, albumin; AST, aspartate aminotransferase; ALT, alanine aminotransferase; γ-GTP, γ-glutamyl transpeptidase; IFA, intrinsic factor antibody; WT1, Wilm’s tumor-1; CHE, cholinesterase; LDH, lactate dehydrogenase; T-bil, total bilirubin; BUN, blood urea nitrogen; Cr, creatinine; UA, uric acid; T-CHO, total cholesterol; TG, triglyceride; Glu, glucose; HbA1c, hemoglobin A1c; Na, sodium; K, potassium; Cl, chloride; CRP, C-reactive protein; Fe, iron; UIBC, unsaturated iron-binding capacity; NCC, nucleated cell count; Mgk, megakaryocyte; Pro-E, proerythroblast; Ba-E, basophilic erythroblast; Orth-E, orthochromatophilic erythroblast; Poly-E, polychromatophilic erythroblasts; Pro-Myelo, promyelocyte; Myelo, myelocyte; Meta-Myelo, metamyelocyte; Stab, stab cell; Seg, segmented neutrophils; Eosino, eosinophil; Baso, basophil; Mono, monocyte; Plasma, plasma cell; MФ, macrophage

Complete Blood Count	Value	Reference Range
WBC	9,600/μL	3,300–8,600/μL
Seg	87%	45–60%
Lymphocyte	9%	25–45%
Mono	4%	4–7%
RBC	152 × 10^4^/μL	386–492 × 10^4^/μL
Hb	6.8 g/dL	11.6–14.8 g/dL
Ht	20.1%	35.1–44.4%
MCV	132.2 fL	83.6–98.2 fL
PLT	9.4 × 10^4^/μL	15.8–34.8 × 10^4^/μL
Urinalysis	Value	Reference Range
Color	Yellow	–
Protein	(+/–)	(–)
Glucose	(–)	(–)
Occult blood	(+)	(–)
Urinary Sediments	Value	Reference Range
Reb blood cell	1–4/H	1–4/H
White blood cell	30–40/H	1–4/H
Serological Test	Value	Reference Range
Haptoglobin	87 mg/dL	19–170 mg/dL
IFA	(+)	(–)
Genetic Test	Value	Reference Range
WT1 mRNA	2.3 × 10^2^ copy/μg RNA	<50 copy/μg RNA
Biochemistry Test	Value	Reference Range
TP	5.6 g/dL	6.6–8.1 g/dL
Alb	3.5 g/dL	4.1–5.1 g/dL
AST	22 U/L	13–30 U/L
ALT	15 U/L	7–23 U/L
ALP	219 IU/mL	38–113 U/L
γ-GTP	13I U/L	9–32 U/L
CHE	181 U/L	240–486 U/L
LDH	378 U/L	124–222 U/L
T-bil	1.0 mg/dL	0.4–1.5 mg/dL
BUN	19.8 mg/dL	8–20 mg/dL
Cr	0.42 mg/dL	0.46–0.79 mg/dL
UA	3.4 mg/dL	2.6–5.5 mg/dL
T-CHO	137 mg/dL	142–248 mg/dL
TG	86 mg/dL	40–234 mg/dL
Glu	142 mg/dL	73–109 mg/dL
HbA1c	4.80%	4.3–5.8%
Na	137 mEq/L	138–145 mEq/L
K	4.5 mEq/L	3.6–4.8 mEq/L
Cl	103 mEq/L	101–108 mEq/L
CRP	6.66 mg/dL	0.00–0.14 mg/dL
Fe	44 μg/dL	80–200 μg/dL
UIBC	126 μg/dL	168–252 μg/dL
Ferritin	439.4 mg/mL	39.4–340 ng/mL
Vitamin B12	57 pg/mL	180–914 pg/mL
Folic acid	2.1 ng/mL	≧4.0 pg/mL
Bone Marrow	Value	Reference Range
NCC	34 × 10^4^/μL	10.0-25.0×10^4^/μL
Mgk	345/μL	50-150×104/μL
Pro-E	0.2%	0.2-1.3%
Ba-E	4.4%	0.5-2.4%
Poly-E	19.8%	17.9-29.2%
Orth-E	1.0%	0.4-4.6%
Myeloblast	0.6%	0.2-1.5%
Pro-mye	2.0%	2.1-4.1%
Mye	13.4%	8.2-15.7%
Meta–myelo	9.8%	9.6-24.3%
Stab	9.8%	9.5-15.3%
Seg	30.2%	6.0-12.0%
Eosino	1.0%	1.2-5.3%
Baso	0.2%	0.0-0.2%
Lymphocyte	0.4%	11.1-23.2%
Mono	0.4%	0.2-4.0%
Plasma	2.2%	0.4-3.9%
MФ	0.6%	0-0.9%
M/E	2.7	1.5-3.3
Chromosome	46, XX	46, XX

**Figure 2:**
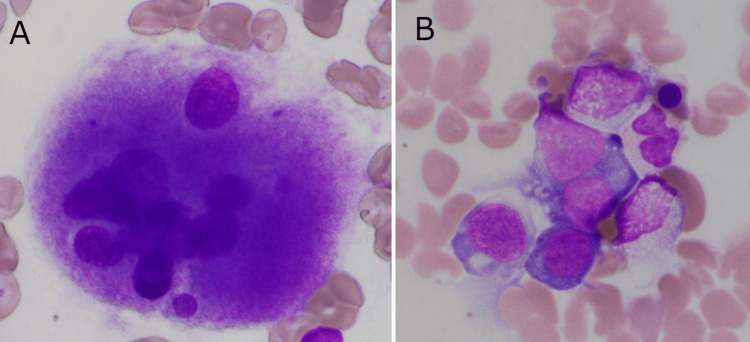
May-Grunwald-Giemsa staining of the patient's bone marrow when she developed resistance to replacement therapy A: Isolated multinucleated megakaryocyte (× 1,000). B: Megaloblastic change (× 1,000).

Replacement therapy with vitamin B12, folic acid, and iron improved the patient’s Hb, MCV, PLT, and WT1 mRNA levels. However, extending the administration interval of vitamin B12 replacement therapy resulted in decreased Hb and PLT, accompanied by an increase in WT1 mRNA. Shortening the interval led to increased Hb and PLT and normalized WT1 mRNA levels. Upon re-examination of her bone marrow aspirate, isolated multinucleated megakaryocytes (Figure 2A), megaloblastic changes (Figure 2B), and a new complex chromosomal abnormality were observed.

Despite a persistently high vitamin B12 concentration, normal MCV, and increasing PLT, the patient’s anemia progressed, and her WT1 mRNA level increased (Table 3).

**Table 3:** Laboratory data when the patient developed resistance to replacement therapy WBC, white blood cell count; RBC, red blood cell count; Hb, hemoglobin; Ht, hematocrit; MCV, mean corpuscular volume; PLT, platelet; TP, total protein; ALB, albumin; AST, aspartate aminotransferase; ALT, alanine aminotransferase; γ-GTP, γ-glutamyl transpeptidase; LDH, lactate dehydrogenase; T-bil, total bilirubin; BUN, blood urea nitrogen; Cr, creatinine; UA, uric acid; Glu, glucose; Na, sodium; K, potassium; Cl, chloride; CRP, C-reactive protein; NCC, nucleated cell count; Mgk, megakaryocyte; Pro-E, proerythroblast; Ba-E, basophilic erythroblast; Poly-E, polychromatophilic erythroblast; Orth-E, orthochromatophilic erythroblast; Pro-Myelo, promyelocyte; Myelo, myelocyte; Meta-Myelo, metamyelocyte; Stab, stab cell; Seg, segmented cell; Eosino, eosinophil; Baso, basophil; Mono, monocyte; Plasma, plasma cell; MФ, macrophage; WT1, Wilm’s tumor-1

Complete Blood Count	Value	Reference Range
WBC	5,300/μL	3,300-8,600/μL
Segmented neutrophil	86.3%	45-60%
Lymphocyte	8.5%	25-45%
Monocyte	4.2%	4-7%
Eosinophil	0.6%	1-4%
Basophil	0.4%	0-1%
RBC	201×10^4^/μL	386-492×10^4^/μL
Hb	6.6g/dL	11.6-14.8 g/dL
Ht	19.3%	35.1-44.4%
MCV	96.2 fL	L83.6-98.2 fL
Plt	24.4×10^4^/μL	15.8-34.8×10^4^/μL
Urinalysis	Value	Reference Range
Color	Brown	-
Protein	1 (+)	(-)
Glucose	(-)	(-)
Occult blood	3(+)	(-)
Urinary Sediments	Value	Reference Range
Reb blood cell	30-49/H	1-4/H
White blood cell	100↑/H	1-4/H
Biochemistry Test	Value	Reference Range
TP	4.1 g/dL	6.6-8.1 g/dL
Alb	1.4 g/dL	4.1-5.1 g/dL
AST	41 U/L	13-30 U/L
ALT	26 U/L	7-23 U/L
ALP	117 IU/mL	38-113 U/L
γ-GTP	17 IU/L	9-32 U/L
LDH	339 U/L	124-222 U/L
T-bil	0.3 mg/dL	0.4-1.5 mg/dL
BUN	43.4 mg/dL	8-20 mg/dL
Cr	0.42 mg/dL	0.46-0.79 mg/dL
Glu	80 mg/dL	73-109 mg/dL
Na	126 mEq/L	138-145 mEq/L
K	3.6 mEq/L	3.6-4.8 mEq/L
Cl	85 mEq/L	101-108 mEq/L
CRP	1.69 mg/dL	0.00-0.14 mg/dL
Ferritin	1978.2 mg/mL	39.4-340 ng/mL
Vitamin B12	33,00 pg/mL	180-914 pg/mL
Folic acid	12.7 ng/mL	>4.0 pg/mL
Genetic Test	Value	Reference Range
WT1 mRNA	64 copy/μgRNA	<50 copy/μg RNA
Bone Marrow	Value	Reference Range
NCC	11.6x10^4^/μL	10.0-25.0×10^4^/μL
Mgk	65/μL	50-150×104/μL
Pro-E	0.2%	0.2-1.3%
Ba-E	1.4%	0.5-2.4%
Poly-E	11.6%	17.9-29.2%
Orth-E	0.2%	0.4-4.6%
Myeloblast	1.4%	0.2-1.5%
Pro-Myelo	1.4%	2.1-4.1%
Myelo	15.6%	8.2-15.7%
Meta-Myelo	12.8%	9.6-24.3%
Stab	13.8%	9.5-15.3%
Seg	32.0%	6.0-12.0%
Eosino	1.2%	1.2-5.3%
Baso	0%	0.0-0.2%
Lymphocyte	5.4%	11.1-23.2%
Monocyte	1.4%	0.2-4.0%
Plasma	1.0%	0.4-3.9%
MФ	0.6%	0-0.9%
M/E	5.9	1.5-3.3
Chromosome	46,XX,?inv(2)(p21q21)	46, XX
	47,XX,t(6:11)(q21:q23.3),+15	

## Discussion

PA is associated with various malignant tumors. The standardized incidence ratio of cancer for patients with PA is the highest in both sexes, with increased risks in the esophagus, stomach, and pancreas. Among hematopoietic tumors, AML is more prevalent in men, whereas multiple myeloma is more prevalent in women [7]. The coexistence of PA and MDS is exceptionally rare, with only one case having been reported in the literature to date. That patient was presumed to have developed PA and MDS concurrently [4]. In our case, anemia responded temporarily to replacement therapy, although it recurred later. The presence of chromosomal abnormalities, megaloblastic changes in the bone marrow, and megakaryocyte dysplasia indicated complications related to MDS. Therefore, this is the second reported case of concurrent PA and MDS. Pancytopenia results directly from impaired DNA synthesis in the progenitor cells caused by vitamin B12 deficiency. MDS is a neoplastic disease characterized by a persistent reduction in red blood cell production and is often associated with a decrease or abnormality in hematopoietic stem cells. The conditions are generally considered mutually exclusive. However, patients with PA may exhibit the characteristic chromosomal abnormalities of MDS, along with similarities in cell dysplasia, suggesting a potential clonal evolution pathway. Despite the presence of chromosomal abnormalities in MBA, replacement therapy reduces the abundance of cells with such abnormalities significantly. Consequently, clonal evolution is unlikely to be the sole cause of MDS in patients with MBA. Although several cases of combined PA and AML have been reported, no definitive relationship has been established between the two, and the combination is often considered coincidental [8,9]. WT1, with an oncogenic role in hematopoietic progenitor cells [10], is a tumor marker that is expressed specifically in MDS and leukemia cells [11]. Quantitative WT1 mRNA testing is therefore used for the therapeutic monitoring of MDS. In a study involving 30 patients with MBA, 10 exhibited WT1 mRNA expression. Although no population-based study has been conducted on WT1 mRNA expression in MBA, its expression in 10 of our 30 cases was considered to represent a relatively high frequency. Among them, two had chromosomal abnormalities common to all myeloid neoplasms, whereas three had chromosomal abnormalities without WT1 mRNA expression. After replacement therapy, WT1 mRNA expression and chromosomal abnormalities disappeared in all the patients who were followed up. This suggests a relationship between MBA pathology and WT1 mRNA expression. However, large-scale retrospective studies are warranted to determine whether WT1 mRNA expression is associated with MBA and MDS. Fanconi anemia is a disease that is mainly caused by chromosomal fragility at the hematopoietic stem cell level and is reported to be associated with leukemia and MDS at a frequency of 13%-22% [12-14]. Hematopoietic stem cells exhibit both self-renewal and multipotency. Their cell division requires DNA synthesis, which depends on active folic acid as a coenzyme. Vitamin B12 is not directly involved in this reaction but plays a role in a biochemical step that prepares the active form of folic acid in the body. Therefore, it has been suggested that the hematopoietic stem cells of MBA also have a DNA duplication disorder that causes pancytopenia and chromosome fragility. However, unlike the case in Fanconi anemia, impaired DNA synthesis in hematopoietic stem cells is typically reversible using replacement therapy - as is the case in MBA. In this case study, replacement therapy temporarily improved anemia and suppressed WT1 mRNA expression, although both the symptoms subsequently recurred, became irreversible, and a new chromosomal abnormality was detected. Our patient with replacement therapy-resistant anemia exhibited a normal MCV, and there was no evidence of hemolysis or thrombocytopenia. This indicates a transition to MDS, but not the recurrence of PA. Dysplastic clones may be established through exposure to further mutations that accelerate tumor cell proliferation, particularly in the context of age-related decline during immune and DNA damage surveillance [15].

Herein, we discuss similarities between MDS and the potential transition to MDS in cases of MBA, based on WT1 mRNA expression. It remains unclear whether WT1 mRNA quantification serves as an early indicator of neoplastic changes in MBA. It is also unclear whether the relationship with MDS is limited to PA, rather than MBA.

The present study has some potential limitations. While the findings are useful, the small sample size and restricting participants to older Japanese adults may not provide enough evidence to identify significant associations between PA and MBA. Additionally, whether the phenomenon is similar in other nationalities and age groups remains uncertain. Therefore, it is necessary to continue accumulating more cases globally and conduct prospective studies on the topic.

## Conclusions

In the present study, we first showed similarities between MBA and MDS in the expression of WT1mRNA. Replacement therapy abolished WT1 mRNA expression as well as chromosomal abnormalities in 100% of the patient cohort. Although WT1 mRNA expression in MBA was also transient, this suggests the presence of cells that may potentially become tumorigenic. However, the mechanism by which these cells progress to irreversible tumor cells requires further investigation. In this study, WT1 mRNA expression was limited to patients with anti-IFA-positive PA. Our patient is the second case of combined PA and MDS.

Retrospective accumulation of cases will be required to determine whether progression to MDS is specific to PA. In cases of PA that have become resistant to treatment, it is important to monitor WT1 mRNA expression and consider the possibility of complications and extension of MDS.
